# Giant cell arteritis initially presents as pulmonary embolism

**DOI:** 10.1515/rir-2026-0004

**Published:** 2026-03-30

**Authors:** Xinyuan Xu, Dan Wang, Tienan Zhu, Jing Li

**Affiliations:** Department of Rheumatology and Clinical Immunology, Peking Union Medical College Hospital, Chinese Academy of Medical Sciences & Peking Union Medical College, Beijing, China; National Clinical Research Center for Rheumatic and Autoimmune Diseases (NCRC-RAD), Ministry of Science & Technology, Beijing, China; State Key Laboratory of Complex Severe and Rare Diseases, Peking Union Medical College Hospital, Beijing, China; Key Laboratory of Rheumatology and Clinical Immunology, Ministry of Education, Beijing, China; Department of Rheumatology and Clinical Immunology, Xi’an Fifth Hospital, Xi’an, Shaanxi Province, China; Department of Hematology, Peking Union Medical College Hospital, Chinese Academy of Medical Sciences & Peking Union Medical College, Beijing, China

Dear Editor,

We describe a rare case of giant cell arteritis (GCA) initially presenting with pulmonary embolism. Given that GCA primarily involves large vessels, pulmonary manifestations as the first presentation are exceptional and may obscure timely diagnosis. This report emphasizes recognizing GCA as a potential cause of unexplained pulmonary embolism and briefly reviews related evidence on its clinical and therapeutic characteristics.

**Figure 1:**
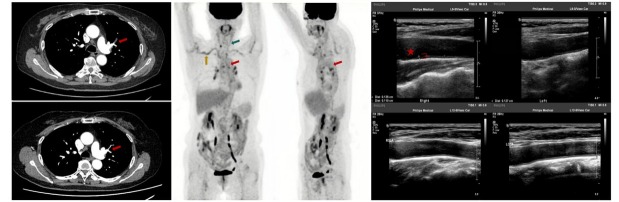
Integrated imaging findings of pulmonary involvement and large-vessel inflammation in GCA. (A) Pulmonary imaging on CTPA before and after treatment. Before treatment (upper panel): CTPA identified a filling defect in the left upper lobe pulmonary artery, consistent with pulmonary embolism; After treatment (lower panel): CTPA demonstrated complete resolution of the pulmonary embolism, with no residual filling defects and only mild stenosis remaining in the upper lobe branches of the left pulmonary artery. (B) PET-CT findings in GCA. The coronal plane (left) and sagittal plane (right) PET-CT images show increased radioactive uptake in the aorta (red arrows), bilateral subclavian and axillary arteries (yellow arrows), and bilateral common carotid arteries (green arrows), consistent with large-vessel inflammation. (C) Vascular ultrasound of the carotid arteries before and after treatment. Before treatment (upper panel): Carotid vascular ultrasound shows diffuse, concentric wall thickening of the common carotid artery, consistent with active large-vessel vasculitis; After treatment (lower panel): Follow-up vascular ultrasound shows marked improvement, with no evident arterial wall thickening after prednisone and tocilizumab therapy.

A 69-year-old woman presented with intermittent hemoptysis for over one year, accompanied by migratory musculoskeletal pain, morning stiffness of the right shoulder, intermittent headaches, and left-sided chest pain. Computed tomography pulmonary angiography (CTPA) detected a left upper lobe pulmonary embolism (Figure 1) with an elevated ventilation/ perfusion ratio, despite normal D-dimer levels and no genetic coagulation abnormalities. Laboratory tests, including autoimmune markers such as antinuclear antibodies, antiphospholipid antibodies, anti-neutrophil cytoplasmic antibodies, as well as inflammatory indicators like erythrocyte sedimentation rate (ESR) and C-reactive protein (CRP), were normal. The absence of deep vein thrombosis on lower extremity ultrasound further supported a non-thrombotic etiology. Given these findings, further evaluations at our center revealed extensive large-vessel inflammation. Positron emission tomography-computed tomography (PET-CT) showed increased metabolic activity in the aorta (maximum standardized uptake value, SUVmax 2.9), and in the brachiocephalic trunk and bilateral subclavian arteries (SUVmax 4.7). Computed tomography angiography (CTA) demonstrated circumferential wall thickening and luminal narrowing involving the aortic arch, thoracic and abdominal aorta, and their branches. Vascular ultrasound confirmed diffuse wall thickening of the axillary, subclavian, and carotid arteries, establishing the diagnosis of GCA.

The 2017 GCA Actemra trial demonstrated that subcutaneous tocilizumab 162 mg weekly or biweekly was an effective and safe therapy. It enabled glucocorticoid tapering and discontinuing within six months and reduced relapse rates compared with glucocorticoid monotherapy.^[1]^ Accordingly, our patient was treated with oral prednisone 40 mg daily, subcutaneous tocilizumab 162 mg administered fortnightly, and oral rivaroxaban 20 mg daily for anti-coagulation. During follow-up, her shoulder pain, morning stiffness, headache, migratory pain, and hemoptysis resolved. Three months later, Vascular ultrasound revealed improvement of the wall thickening in the axillary, subclavian, and carotid arteries. After nine months, following prednisone discontinuation, repeated CTA demonstrated resolution of arterial wall thickening and luminal stenosis, and CTPA indicated resolution of pulmonary embolism, leaving mild stenosis in the upper lobe branches of left pulmonary artery. Overall, these improvements indicate that tocilizumab effectively suppresses vascular inflammation and achieves sustained glucocorticoid-free remission in GCA.

A review of the literature identified six reported cases of GCA that initially manifested as pulmonary embolism, including our case (Table 1). Respiratory symptoms were the predominant initial manifestations and frequently contributed to diagnostic delay due to their nonspecific nature. Earlier cases were confirmed through histopathological examination, whereas more recent reports have increasingly relied on advanced imaging techniques to detect large-vessel inflammation. The majority of individuals achieved complete remission with corticosteroid therapy.

**Table 1:** Reported cases of giant cell arteritis initially presents as pulmonary embolism.

Authors	Year	Age	Sex	Initial Clinical Manifestations	Diagnostic methods	Treatment	Outcome
Doyle *et al*.^[7]^	1988	67	Male	Cough, yellow sputum, and chest pain	Histopathology of the resected lung lobe	Steroids	Deterioration
Radhamanohar^[8]^	1991	83	Female	Dyspnoea, weight loss, malaise, and headaches	temporal artery biopsy	Prednisolone	Complete remission
Andrès *et al*^[9]^	2004	80	Female	Dyspnoea	Post-mortem anatomopathological examination	Not reported	Death
Shu *et al*^[6]^	2017	62	Male	Fever, body aches and swollen feet	PET-CT	Methylprednisolone	Complete remission
Gonçalves *et al*^[10]^	2022	79	Female	Left thoracic pain and temporal headache	CTPA	Prednisolone	Complete remission
Our case	2025	69	Female	Hemoptysis and left-sided chest pain	CTPA, CTA, vascular ultrasound and PET-CT	Prednisone and tocilizumab	Complete remission

PET-CT, positron emission tomography-computed tomography; CTPA, computed tomography pulmonary angiography; CTA, computed tomography angiography.

Notably, while inflammatory indicators (such as ESR and CRP) are typically elevated in active GCA,^[2]^ approximately 1–2% of individuals exhibit normal or mildly increased levels, potentially leading to oversight of vasculitis.^[3]^ In the reported cases, inflammatory indicators were elevated in some cases but normal in others. In our case, the woman also had normal level of inflammatory indicators. The study on the correlation between inflammatory indicators and GCA showed that the mean value of ESR in patients with severe manifestations of ischemia was significantly lower than that in patients without (*P* = 0.001), which suggests that inflammatory indicators are not absolutely correlated with the disease activity in GCA.^[4]^

Currently, the mechanisms of *in situ* pulmonary arterial thrombosis associated with GCA remain incompletely understood. It is likely the result of a multifactorial process involving systemic inflammation that induces hypercoagulability, vascular endothelial injury, and local blood flow stasis.^[5]^ Active inflammation promotes procoagulant factor expression, suppresses natural anticoagulant and fibrinolytic pathways, and enhances platelet activation, thereby amplifying thrombus formation.^[5]^ GCA is characterized by granulomatous inflammation of medium- and large-sized arteries, which disrupts the vascular wall and endothelial integrity, reduces the production of antithrombotic mediators such as nitric oxide and prostacyclin, and exposes subendothelial procoagulant surfaces.^[5]^ Additionally, intimal hyperplasia and luminal narrowing may alter hemodynamics, providing a structural basis for *in situ* thrombosis.^[5]^

Collectively, these mechanisms underscore the importance of heightened clinical vigilance and suggest that early anti-inflammatory and individualized anticoagulation strategies may help prevent vascular complications in GCA. Although rare, GCA can cause pulmonary thromboembolism even when inflammatory indicators are normal. For patients with suspected large-vessel involvement lacking temporal or cranial symptoms, PET-CT represents a valuable modality for detecting occult vascular inflammation,^[6]^ whereas temporal artery biopsy continues to serve as the gold standard of diagnosis.
